# Impact of porous microsponges in minimizing myotoxic side effects of simvastatin

**DOI:** 10.1038/s41598-023-32545-0

**Published:** 2023-04-08

**Authors:** Ahmed U. Ali, Mahmoud Abd-Elkareem, Amira A. Kamel, Nasser S. Abou Khalil, D. Hamad, Nasr Eldin Hussein Nasr, Maha A. Hassan, Tahani H. El Faham

**Affiliations:** 1 Department of Pharmaceutics, Faculty of Pharmacy, Merit University, Sohag, Egypt; 2 Department of Cell and Tissues, Faculty of Veterinary Medicine, Assiut University, Assiut, Egypt; 3 Department of Medical Biochemistry and Molecular Biology, Faculty of Medicine, Assiut University, Assiut, Egypt; 4 Department of Medical Physiology, Faculty of Medicine, Assiut University, Assiut, Egypt; 5 Department of Physics, Faculty of Science, Assiut University, Assiut, Egypt; 6 Technical Manager at Al Esraa Pharmaceutical Optima, Badr City, Cairo Egypt; 7 Department of Pharmaceutics, Faculty of Pharmacy, Assiut University, Assiut, Egypt

**Keywords:** Drug discovery, Physiology

## Abstract

Simvastatin (SV) is a poorly soluble drug; its oral administration is associated with a significant problem: Myopathy. The present study aims to formulate SV microsponges that have the potential to minimize the myotoxicity accompanying the oral administration of the drug. SV microsponges were prepared by exploiting the emulsion solvent evaporation technique. The % entrapment efficiency (%EE) of the drug approached 82.54 ± 1.27%, the mean particle size of SV microsponges ranged from 53.80 ± 6.35 to 86.03 ± 4.79 µm in diameter, and the % cumulative drug release (%CDR) of SV from microsponges was significantly higher than that from free drug dispersion much more, the specific surface area of the optimized microsponges formulation was found to be 16.6 m^2^/g revealed the porosity of prepared microsponges. Histological and glycogen histochemical studies in the skeletal muscles of male albino rats revealed that microsponges were safer than free SV in minimizing myotoxicity. These findings were proven by Gene expression of Mitochondrial fusion and fission (Mfn1) & (Fis1) and (Peroxisome proliferator-activated receptor gamma co-activator 1α) PGC-1α. Finally, our study ascertained that SV microsponges significantly decreased the myotoxicity of SV.

## Introduction

Cardiovascular diseases (CVDs) stand as the leading cause of death worldwide. Atherosclerosis is a fundamental reason for vascular death in which fatty streaks in arterial walls gradually develop into atheroma and plaques, leading to atherosclerosis. There are many reasons for atherosclerosis; hypercholesterolemia is considered the initial factor that allows the operation of other risk factors^1^. Statins hinder the hydroxy-methylglutaryl-coenzyme A (HMG-CoA) reductase enzyme, which is accountable for the first step in sterol biosynthesis leading to a severe diminution of low-density lipoprotein cholesterol (LDL-C)^2^. It also exerts some pleiotropic effects due to reduced production of mevalonate’s isoprenoid products, including decreasing vascular inflammation and oxidative stress while enhancing the stability of atherosclerotic lesions^3^. Statins also reduce the plasma concentrations of tumor necrosis factor-α (TNF-α), improve endothelial function, and minimize mortality and morbidity^4^. Oral administration of SV as a lipid-lowering drug is associated with skeletal muscle-associated symptoms (SAMS), including weakness, fatigue, muscle pain, myopathy, myalgia, and myositis. The most severe reaction is fatal rhabdomyolysis; oral administration of SV as a lipid-lowering drug is associated with skeletal muscle-associated symptoms (SAMS), including weakness, fatigue, muscle pain, myopathy, and myositis. Additionally, the levels of a muscle enzyme called Creatine kinase (CK) are elevated^5^. Peroxisome proliferator-activated receptor gamma co-activator (PGC-1α) is a co-activator of transcription. It controls mitochondrial biogenesis and function, oxidative phosphorylation, and the detoxification of reactive oxygen species^6^. In metabolically active tissues such as skeletal muscle, PGC-1α is highly expressed^7^. Its expression is reduced when mitochondrial biogenesis is disrupted. Mitochondria are highly dynamic organelles constantly moving and undergoing fusion and fission. Mitochondrial fusion and fission (MFF)-related proteins, including Mfn1 and Fis1, regulate these processes. Mitochondria maintain a balance between fusion and fission to control their shape and function. Several changes occur when this balance is disrupted, and the expression levels of the above proteins alter^8^. Microsponges are porous polymeric microspheres with interconnecting pores in their structure. They offer many advantages, including easy preparation, large-scale production, and high stability at elevated temperatures up to 130 °C. In addition to these desirable attributes, microsponges can entrap various drugs, including hydrophilic and hydrophobic drugs, and control drug release. In previous work, the selected microsponges formulation was dispersed into different gel bases, and a clinical study was performed on human subjects to treat periodontal defects. Compared to those made with free SV, chitosan 2% gels containing microsponges of SV demonstrated a substantial decrease in both clinical attachment loss and pocket depth^9^. Therefore, the present study aims to formulate SV microsponges to minimize the myotoxic side effect of SV in rats by measuring serum creatine kinase levels and skeletal muscle gene expression levels of PGC-1α, Mfn1, and Fis1, along with histological and immunohistochemical changes in skeletal muscles of rats.

## Methods

### Chemicals

SV was a thoughtful present supplied by Amrita Pharmaceutical Industries, Alexandria, Egypt. Eudragit RS-100 was bought off (Rohm Pharm. Co., Weiterstadt, Germany). Magnesium stearate (Mg stearate) was bought off (Leochem Pvt. Ltd. India). Acetone, methanol, n-hexane, and light liquid paraffin were purchased from (El Nasr Pharmaceutical Chemicals Co., Egypt). All chemicals used were of analytical grade used without further purification.

### Preparation of SV-loaded microsponges

The emulsion-solvent evaporation technique was utilized for the preparation of microsponges^10^. In brief, Eudragit RS-100 and SV were dissolved in a mixture of methanol and acetone at a ratio of 1:5. Then, Mg stearate was dispersed in the former solution using a bath sonicator for about 2 min. The formed dispersion was then poured into a beaker containing 200 ml of light liquid paraffin. The system was stirred using a mechanical stirrer at a speed of 500 rpm for 8 h until acetone and methanol had evaporated entirely. The formed SV microsponges were filtered through filter paper, washed three times with 50 ml *n*-hexane, and dried in a desiccator under reduced pressure (< 15 mmHg) overnight. Eight formulations have been prepared using 2^3^ full factorial designs with three independent variables; each changed at two levels. The three independent variables were (A) the total volume of solvents (acetone and methanol mixture) (12 and 18 ml), (B) the concentration of Mg stearate (0.5 and 1% W/V), and (C) the ambient temperature while stirring (25 and 40 °C). The effect of these independent variables was studied on the dependent factors: the percent entrapment efficiency (% EE), the particle size, and the % cumulative drug released (% CDR). The volume of light liquid paraffin (200 ml), the amount of drug (250 mg), the amount of Eudragit RS-100 (750 mg), and the stirring rate (500 rpm) were kept constant. The compositions of different formulae are illustrated in (Table 1).

**Table 1:** Composition, %EE, PY, particle size, and PDI of different microsponges formulations.

Code	Mg stearate concentration %W/V	Ambient temperature °C	Volume of solvents ml	% EE	PY (%) (mean ± SD)	Particle size (µm) ± SD
FSM-1	0.5	25	18	77.32 ± 2.03	88.53 ± 3.42	86.03 ± 4.79
FSM-2	0.5	40	18	47.62 ± 3.15	77.98 ± 3.68	79.30 ± 3.25
FSM-3	1	25	18	58.32 ± 1.59	76.69 ± 1.98	74.70 ± 6.23
FSM-4	1	40	18	75.00 ± 4.32	70.76 ± 2.36	60.97 ± 10.21
FSM-5	0.5	25	12	58.96 ± 3.65	71.69 ± 1.54	79.46 ± 7.12
FSM-6	0.5	40	12	77.40 ± 2.15	72.16 ± 2.47	53.80 ± 6.35
FSM-7	1	25	12	82.54 ± 1.27	81.68 ± 3.12	65.21 ± 5.42
FSM-8	1	40	12	68.54 ± 3.89	76.33 ± 2.95	65.31 ± 4.25

### Characterization of the prepared microsponges

Production yield (% PY) was determined according to this equation

1 $$\% {\text{ PY = }}\left( {\left( {{\text{Weight of obtained drug}}/{\text{Theoretical weight of drug and polymer}}} \right) \times {1}00} \right).$$

The %(EE) was determined as follows; 80 mg of the developed microsponges were distributed throughout 100 ml of phosphate buffer pH (6.8) and stirred using a magnetic stirrer for 24 h; then, the solution was filtered, and the drug content was quantified spectrophotometrically at 238 nm against a blank, and the %EE was calculated as^11^

2 $$\% {\text{EE }} = \, \left( {\left( {{\text{Actual drug content}}/{\text{Theoretical content}}} \right) \times {1}00} \right).$$

The particle size and particle size distribution of different microsponges formulations were ascertained using a laser scattering particle size distribution analyzer (HORIBA LA-300). Regarding the in-vitro release studies, calculated amounts of the SV microsponges formulations (equivalent to 5 mg of solid SV) were subjected to in vitro release studies; 5 mg of free SV was regarded as a control. The dissolution test was performed in USP rotating paddle apparatus (ERWEKA DT 720) at a stirring rate of 50 rpm at 37 ± 0.5 °C for 24 h. Initially, the release of the drug was carried out in 500 ml of 0.1 N hydrochloric acid pH 1.2 for 2 h. Then, the pH was shifted to 6.8 by adding 200 ml of 0.1 M tribasic sodium phosphate dodecahydrate (TBS) for the next 22 h. Samples were pulled out at definite time intervals: 0.5, 1, 2, 4, 6, 8, 10, 12, and 24 h and were replaced with an equal volume of fresh medium to keep up the sink conditions. The withdrawn samples were analyzed spectrophotometrically for SV at λ_max_ of 238 nm. Data from release studies were fitted to different release models (zero, first, and Higuchi diffusion) to determine the model which describes the kinetic release of SV from the prepared microsponges formulations. The optimized microsponges formulation has been examined using a scanning electron microscope (SEM, JEOL 5400, Tokyo, Japan) to display its surface morphology. For investigating the Powder X-ray diffraction (XRD), CuKα radiation (λ = 1.546 Å) was used in an X-ray Philips Type PW 1710 diffractometer to study the crystal structure in the range of 4°–60°. JCPDS-ICDD is used for analyzing the XRD phases. The nitrogen adsorption–desorption isotherms of the optimized formulation were recorded using the BET technique at 77 K on a Nova-3200 BET surface area analyzer. Before nitrogen adsorption, all materials were dehydrated and outgassed at 150 K for 2 h. After analyzing the desorption branch in the isotherm, the pore size distributions were plotted using the BJH technique and the equipment software. The nitrogen adsorption/desorption technique was used to determine the surface area for the optimized formulation. The total surface area, S_total_, and hence the specific surface area, SBET, of the powder sample were estimated from the BET plot using Eq. (3).

3 $${\text{S}}_{{{\text{tol}}}} = {\text{N}}_{{\text{a}}} {\text{N}}_{{\text{m}}} \sigma ,$$

where Na is Avogadro’s number (6.022 × 1023 mol^–1^), Nm is the number of moles of adsorbate in a monolayer, and σ is the cross-sectional area of the adsorbate molecule.

### Histological and biochemical studies of SV-loaded microsponges in rats

To investigate the effect of the prepared SV microsponges formulations on the skeletal muscles of male albino rats, histological studies were conducted using male albino rats. Thirty-two healthy male rats weighing 200 ± 20 g were used by the standard rules of the Committee of Animal Care, Assiut University, Assiut, Egypt. Animals were kept fasting for 12 h before the experiment with open access to water. The twenty rats were split up into four groups; the first group was held as a control (control group), the second group (free SV group) received free SV, the third (FSM-1 group), and the fourth (FSM-6 group) group received formulae (FSM-1 & FSM-6) respectively. All rats were given a 20 mg/kg dosage once a day of free or its equivalence of microsponges loaded SV suspended in 1% hydroxypropylmethylcellulose via oral gavage needle for 15 days. Heparinized capillary tubes were used to collect blood samples from the retro-orbital plexus of veins. Afterward, it was permitted to clot at room temperature for 20–30 min. The samples were centrifuged for 20 min at 2000 rpm to remove the clot and separate the serum sample, which was kept at − 20 °C until the assay. Finally, the animals were sacrificed by overdosing sodium pentobarbital via intraperitoneal injection. The skeletal muscles (gastrocnemius muscle) were removed and separated into two parts: one for RNA extraction and quantitative Real-Time Polymerase Chain Reaction (qRT-PCR), the other for histological assessment. Histological studies were performed: formalin-fixed samples were dehydrated in ascending grades of ethanol, cleared in methyl benzoate, and embedded in paraffin wax. Paraffin sections of 5 µm in thickness were cut and stained with the following histological stains: Haematoxylin and Eosin (H&E) for general histological examination, Periodic acid Schiff (PAS) technique for the histochemical demonstration of glycogen^12^, Masson’s trichrome method to stain collagen fibers, Sirius red approach differentiates between mature and immature collagen^13^ and Silver impregnation technique for demonstration of cross (transverse) striation of myofibrils. An Olympus BX51 microscope examined all staining preparations, and the photographs were taken by an Olympus DP72 camera adapted onto the microscope.

### Negative image analysis using CMEIAS color segmentation

Negative image analysis was performed using CMEIAS color segmentation to assess the complex color micrographs obtained to give more details^14–16^.

### Immunofluorescence of glutathione reductase (GR) and superoxide dismutase 2 (SOD2) in the skeletal muscles

Polyclonal anti-glutathione reductase, anti-superoxide dismutase 2 antibodies (Chongqing Biospes Co., Ltd, China), and Goat anti-Mouse IgG2A Secondary Antibody [FITC] (Novus Biologicals, USA) were utilized for the immunohistochemical detection of glutathione reductase (GR) and superoxide dismutase 2 (SOD2) in the skeletal muscles. 5 µm sections of paraffin-embedded tissues were deparaffinized by xylene and rehydrated using a descending series of ethanol and then double-distilled water. Slides were then rinsed in PBS at a pH of 7.4 (three times for one minute each time). The slides were located in a jar containing 100 ml 0.1 M citrate buffer, pH 6.0, and heated to near boiling (95–98 °C) in a water bath for 30 min, followed by cooling for 20 min at 25 °C. Sections were then rinsed in PBS at a pH of 7.4 (three times for 1 min each time). The slides were then incubated with anti-glutathione reductase and anti-superoxide dismutase 2 antibodies in a dilution of (1:100) overnight in a humidified atmosphere at room temperature. The slides were then washed with PBS pH 7.4 (3 times) and then incubated for 1 h with Goat anti-Mouse IgG2A Secondary Antibody [FITC, NB7515] in a humidified chamber at 37 °C in the dark. Slides were washed three times with PBS and analyzed under a fluorescence microscope.

Serum creatine kinase (CK) was measured via enzymatic colorimetric method using commercially available kits (BioVision creatine kinase Activity Colorimetric Assay Kit, catalog # K777-100 USA) according to manufacturer instructions. All experimental protocols were approved by the ethical committee of Faculty of Pharmacy, Assiut University, Assiut, Egypt, Approval no S 14–21, and were performed according to ARRIVE guidelines of animal experiments. All methods were performed in accordance with the relevant guidelines and regulations.

### RNA extraction and real-time quantitative PCR (qRT-PCR)

Following the kit instructions, total RNA was isolated from muscle tissue samples using ThermoFisher Scientific GeneJET RNA Purification Kit Catalog number: K0731. ThermoFisher Scientific’s High-Capacity cDNA Reverse Transcription Kit Catalog number: 4368814 was used for reverse transcriptase activities. The following conditions were used to amplify cDNA: 95 °C for 10 min, then 40 cycles of denaturation at 95 °C for 15 s, annealing at 60 °C for 20 s, and extension at 72 °C for 40 s, with data collected in the final 30 s. The PowerUpTM SYBRTM Green Master Mix, Catalog number A25779, from ThermoFisher Scientific, is used in this reaction. Real-time PCR was carried out using Applied Biosystems StepOnePlus software. The levels of reference gene β-Actin normalized levels of transcription of target genes. Relative gene expression levels of all examined genes were measured using the comparative 2-ΔΔCT ^17^.

Primer sequences of PGC-1α, Mfn1, Fis1, and β-actin used were:

- PGC-1α for 5-GCACCAGAAAACAGCTCCAA-3;
- PGC-1α rev 5-TTGCCATCCCGTAGTTCACT-3;
- Mfn1 for 5-CGCCTGTCTGTTTTGGTTGA-3;
- Mfn1 rev 5-GCATTGACTTCACTGGTGCA-3;
- Fis1 for 5-AAAGAGGAGCAGCGGGATTA-3;
- Fis1 rev 5-TGGGGCTCAGTCTGTAACAG-3;
- β-Actin for 5-TCTTCCAGCCTTCCTTCCTG-3;
- β-Actin rev 5-CAATGCCTGGGTACATGGTG-3.

### Statistical analysis

The data were presented as a mean ± SD. One-way analysis of variance was used to determine statistical significance, followed by an LSD post hoc test. Pearson’s correlation coefficients were used to examine correlations. The significance was considered at P values < 0.05. The results were analyzed using SPSS 20.

## Results and discussion

**Figure 1:**
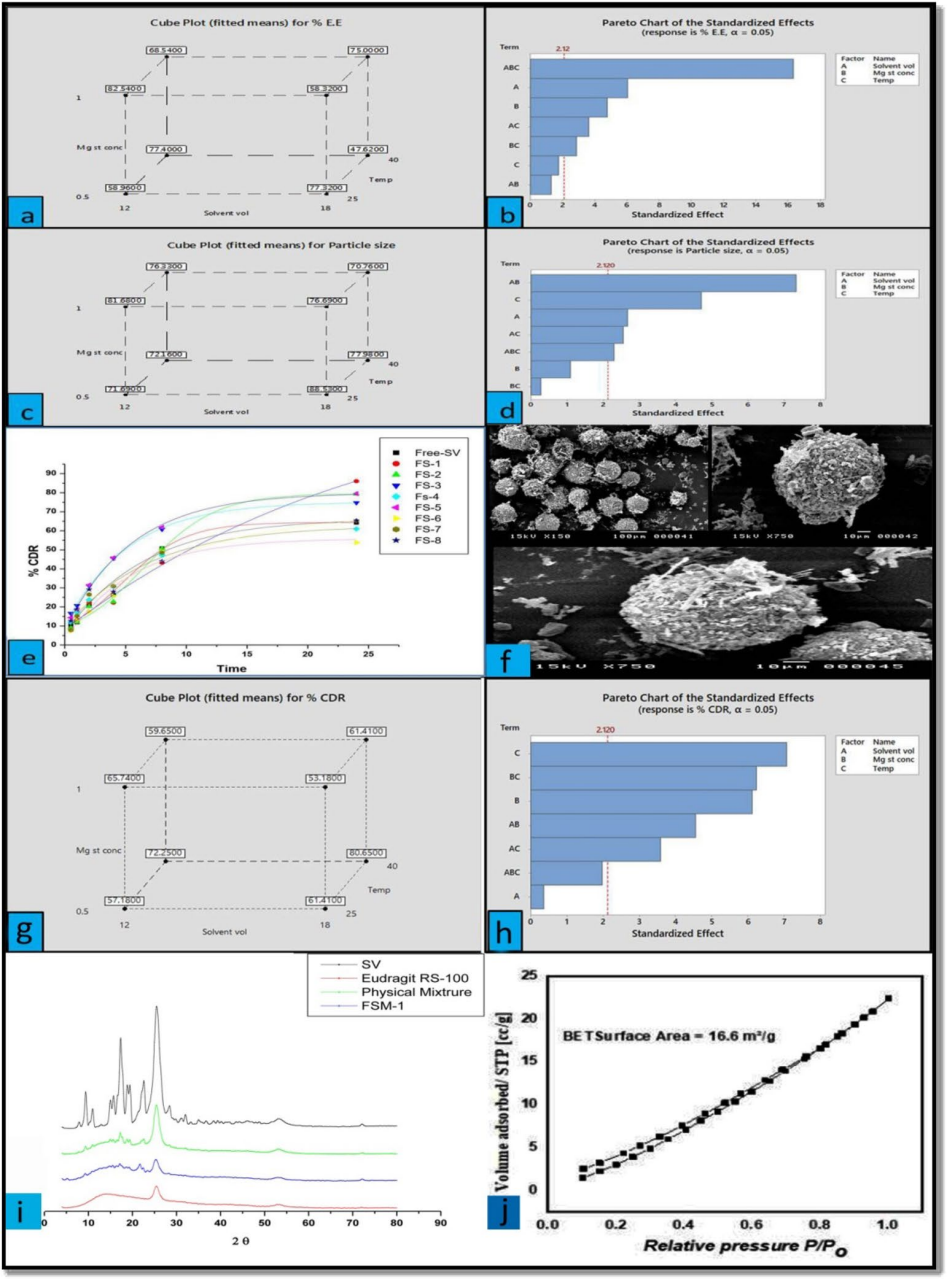
(**a**) Cube plot (fitted means) for %EE. (**b**) Pareto chart of standardized effects (response is %EE. (**c**) Cube plot (fitted means) for particle size. (**d**) Pareto chart of standardized effects (response is particle size). (**e**) In vitro release profile of SV from different micrsoponges formulations and free SV dispersion. (**f**) Cube Plot (fitted means) for % CDR. (**g**) Pareto chart of standardized effects: response is %CDR. (**h**) SEM of optimized formulation of SV microsponges. (**i**) X-ray diffraction pattern for SV, Eudragit-RS-100, Physical mixture, and FSM-1. (**j**) N2 adsorption–desorption hysteresis of FSM-1.

The PY of the prepared microsponges formulations was so high (70.76 ± 2.36–88.53 ± 3.42), indicating the efficiency of the preparation method. The % EE of prepared microsponges formulae was calculated and summarized in (Table 1). It was observed that decreasing the solvent volume and increasing the concentration of Mg stearate resulted in increasing the % EE (Fig. 1a). The enhancement of the % EE on decreasing the solvent volume may be due to a rise in the viscosity of the internal phase, which hindered the diffusion of acetone so that more time is needed for the formation of larger droplets containing more significant amounts of the drug^18^ while, on increasing the concentration of Mg stearates from 0.5 to 1%, the % EE increased which could be assigned to the stabilization effect of Mg stearate on the formed droplets; however, there are some exceptions. FSM-3 had a higher concentration of Mg stearate but showed a lower %EE than FSM-1, with a lower Mg stearate concentration. The same was observed between FSM-6 and FSM-8. This exception may be attributed to two facts; the first is that not all the amount of Mg-stearate is incorporated into the microsponges^19^. In addition, some SV may be lost during preparation or leaking via diffusion into liquid paraffin^20^. Besides that, another explanation was proposed by Yüksel and Baykara who suggested that the drug may leak through the porous structure of the microspheres^21^. The effect of the three independent factors on %EE was presented in (Fig. 1a). Two-way ANOVA revealed that the impact of the three independent factors (the total volume of solvents, the concentration of Mg stearate, and the ambient temperature during stirring) separately was highly significant (*P* < 0.005) on the %EE. Identical results were obtained in the two- and three-way interactions except for the combined effect of solvent volume and Magnesium st concentration which was insignificant on the %EE (Fig. 1b). The regression equation of %EE^22^

4 $$\% {\text{EE }} = - \;{594}.{5} - {7}.{\text{295 Solvent}} {\text{vol }} + {5}.{775} {\text{Mg st conc}} - {2}.{\text{145 Temp 1}}.{\text{585 Solvent vol}} \times {\text{Mg st conc}} - {4}.{365}\; {\text{Solvent vol}} \times {\text{Temp }} + { 3}.{485} {\text{Mg st conc}} \times {\text{Temp }} + {19}.{7}0{5} {\text{Solvent vol}} \times {\text{Mg st conc}} \times {\text{Temp}},$$

The particle size and particle size distribution of different microsponges formulae were cited in (Table 1).

It was observed that the particle size reduced on raising the Mg stearate concentration (Fig. 1c). Mg-stearate is adsorbed onto the droplet surface, decreasing the interfacial tension so that the particle size decreases by increasing the Mg stearate concentration^20^. Moreover, increasing the concentration of Mg stearate speeds up the dispersion of microspheres in the system. The influence of preparation temperature was studied on the particle size. Results revealed that on raising the preparation temperature from 25 to 40 °C, the particle size of the microsponges rose (Fig. 1c); this is attributed to increasing the temperature from 25 to 40 °C. The viscosity of the external phase decreased, resulting in the merging of the droplets as the rate of drainage of the film enhanced and larger particles were obtained. These results align with those obtained by Heiskanen et al. They studied the effect of temperature on the particle size of prepared microspheres using an emulsion–solvent extraction technique^23^. The effect of the three independent factors on particle size was presented in Fig. 1. Two Way ANOVA revealed that the effect of the Mg stearate conc and the combined effect of both Mg stearate conc & temperature were insignificant (*p* < 0.05) on the particle size (Table 1, Fig. 1d). The impact of the three independent factors on particle size was presented in the Pareto chart (Fig. 1d). The regression equation of Particle size^22^

5 $${\text{Particle size }} = - \;{97}.{7 } + {1}.{512}\;{\text{Solvent vol}} - {1}.{\text{225 Mg st conc}} - {5}.{34}0{\text{ Temp}} - {8}.{3}0{\text{5 Solvent vol}} \times {\text{Mg st conc}} - {2}.{9}00{\text{ Solvent vol}} \times {\text{Temp}} - {3}.00 {\text{Mg st conc}} \times {\text{Temp }} + {2}.{61} \;{\text{Solvent vol}} \times {\text{Mg st conc}} \times {\text{Temp}}.$$

The release of SV from different SV microsponge formulations and free SV suspension was investigated and cited in (Fig. 1e). The % CDR of SV from the formulated microsponges ranged from 53.80 ± 2.19 to 86.03 ± 2.24%, while that from free SV suspension after 24 h was only 64.19%. FSM-1 exhibited significantly higher (*P* < 0.05) %CDR than that of the free drug suspension. According to (Fig. 1g), it was found that the % CDR increased as the volume of the internal phase increased, and the concentration of Mg stearate decreased. Increasing the solvent volume from 12 to 18 ml decreases the viscosity of the internal phase. Smaller particles are produced, and it was reported that the dissolution rate is enhanced as the particle size is reduced as a result of increasing the surface area^24^. On the contrary, increasing Mg stearate concentration leads to its accumulation on the surface of the microsponges, which decreases the %CDR due to its hydrophobic nature^25^. A statistical study of data revealed that the effect of solvent volume and the combined effect of all three independent factors on the %CRD was insignificant (P < 0.05). In contrast, the impact of other factors and their combinations was significant (P > 0.05) (Fig. 1h). Two-way ANOVA revealed that the effect of the solvent volume and the combined effect of both Mg stearate conc & Temp was insignificant (*P* < 0.05) on the %CDR (Fig. 1g). The regression equation of %CDR^5^,

6 $$\% {\text{CDR}} = {344}.{5 } + 0.{457}\; {\text{Solvent vol}} - {7}.{\text{877 Mg st conc}} - {9}.{113} {\text{Temp}} - {5}.{857} {\text{Solvent vol }} \times {\text{ Mg st conc }} + {4}.{622} {\text{Solvent vol }} \times {\text{ Temp}} - 0.{8}.0{42} {\text{Mg st conc }} \times {\text{ Temp }} + {2}.{537} {\text{Solvent vol}} \times {\text{ Mg st conc }} \times {\text{ Temp}}).$$

Kinetic investigation of the release data (Table 2) showed that the release profile of SV from the microsponges formulations exhibited zero-order release mechanisms. Using the (Minitab 17.3.1. software), FSM-1 has the smallest particle size of 86.03 ± 4.79 µm, maximum entrapment efficiency of 77.32 ± 2.03%, % CDR of 86.03 ± 1.32%, and desirability factor of 0.8415 was discovered to be the most effective formulation. It was selected for further characterization as well as for the histological study.The morphology and shape of the SV-loaded microsponges that have been optimized were examined by SEM (Fig. 1f). SV microsponges were found to be globular in form and homogeneously distributed. The XRD patterns of Pure SV, Eudragit RS-100, the physical mixture of both, and the optimized microsponges formulation (FSM-1) were depicted in (Fig. 1i). Pure SV is a highly crystalline powder showing distinctive peaks at 14.005, 16.32, 25.75, and 02θ. The intensity of these diffraction peaks was minimized in the diffractogram of the physical mixture of SV and Eudragit RS-100. In contrast, in the diffractogram of the optimized microsponges formulation (FSM-1), the distinctive peaks of pure SV drastically lowered, indicating a significant reduction of the crystallinity of SV.

**Table 2:** Kinetic variables for in-vitro release of SV from different microsponges formulations.

Formula	Correlation coefficient (*R*2)		
	Zero order kinetic mechanism	First order kinetic mechanism	Diffusion order kinetic mechanism
Free SV	0.968002	0.889735	0.937272
FSM-1	0.959706	0.956803	0.932106
FSM-2	0.996264	0.994136	0.981666
FSM-3	0.991890	0.954506	0.951919
FSM-4	0.986878	0.952425	0.951664
FSM-5	0.988906	0.956585	0.934764
FSM-6	0.985177	0.778769	0.979042
FSM-7	0.990835	0.923223	0.984581
FSM-8	0.986487	0.921448	0.928144

### Estimation of surface area

The nitrogen adsorption/desorption technique was used to determine the surface area for the optimized microsponges formulation (FSM-1) powder sample, as presented in (Fig. 1j). The specific surface area of samples was determined using Eq. (3) and was found to be 16.6 m^2^/g. It was found that the surface area is the internal surface area per unit of pore volume and figures out the amount of space in rocks exposed to the injectant during an injection operation.

### Histological study

**Figure 2:**
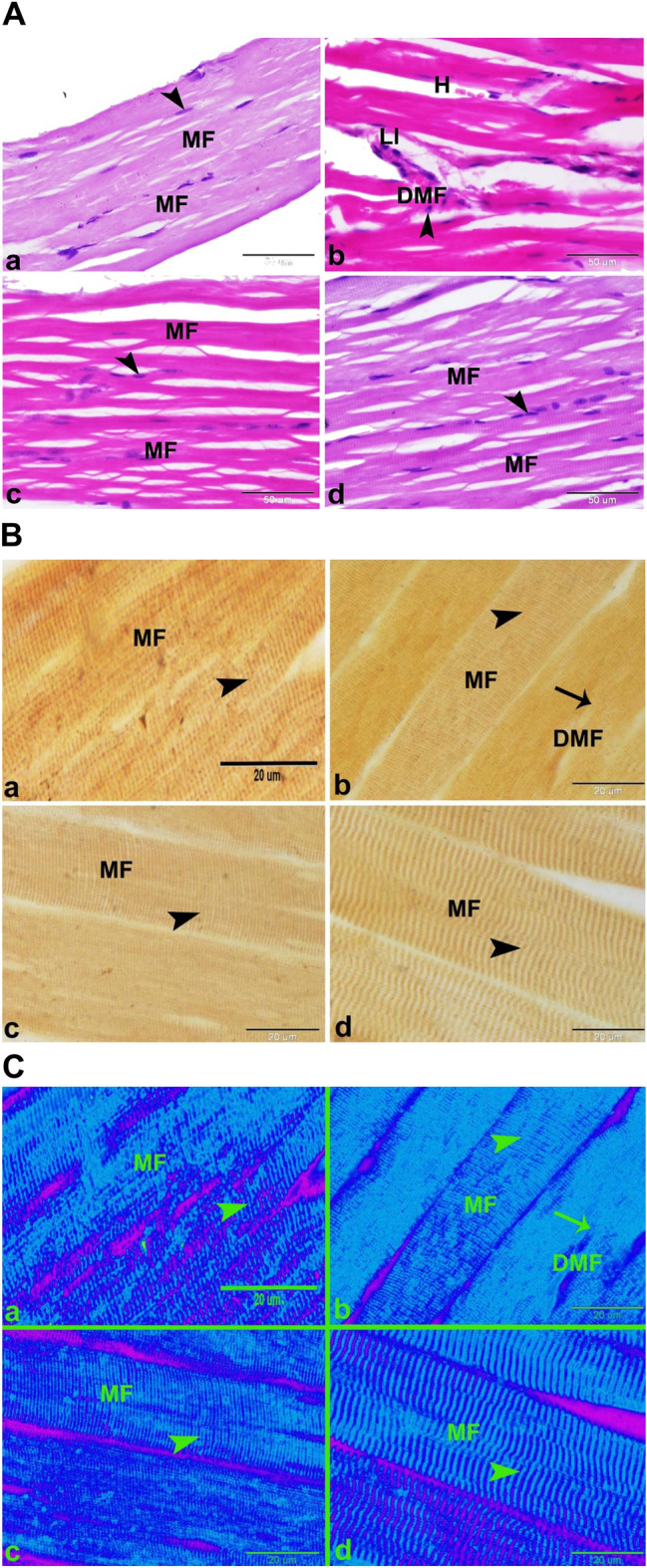
(**A**) Photomicrographs showing minimizing of the myotoxic side effects of SV by using microsponges formulas in rats. (**a**) Control group showing the normal histological structure of the skeletal muscle in rat. The skeletal muscle was formed of several parallel elongated cylindrical muscle fibers (MF) with multiple flattened peripherally located nuclei (arrowhead) beneath the sarcolemma and acidophilic sarcoplasm. (**b**) Free SV group showing the myotoxic side effects of SV as; degenerated muscles fibers with deeply stained acidophilic sarcoplasm, pyknotic nucleus (arrowhead), hemorrhage (H) and leucocytic infiltration (LI). (**c**) FSV-6 group showing slight minimizing of the myotoxic side effects of SV by using microsponges formulation FSV-6. Note the deeply stained skeletal muscle fibers (MF) with multiple flattened peripherally located nuclei (arrowhead) beneath the sarcolemma. (**d**) FSV-1 group showing minimizing of the myotoxic side effects of SV by using microsponges formulation FSV-1.The skeletal muscle was formed of several parallel elongated cylindrical muscle fibers (MF) with acidophilic sarcoplasm and multiple flattened peripherally located nuclei (arrowhead) beneath the sarcolemma .Hx&E, scale bar = 50 μm. (**B**) Photomicrographs showing minimizing of the myotoxic side effects of SV by using microsponges formulas in rats. (**a**) Control group showing the normal skeletal muscle fibers (MF) with clear transvers striations (arrowhead). (**b**) Free SV group showing the myotoxic side effects of SV as; degenerated skeletal muscle fibers (DMF) with absence or ill clear transvers striations (arrowhead). (**c**) FSV-6 group showing skeletal muscle fibers (MF) with ill clear transvers striations (arrowhead). (**d**) FSV-1 group showing minimizing of the myotoxic side effects of SV. Note the skeletal muscle fibers (MF) with clear transvers striations (arrowhead). Silver impregnation technique, scale bar = 50 μm. (**C**) Negative images of the photomicrographs shown in (**B**).

**Figure 3:**
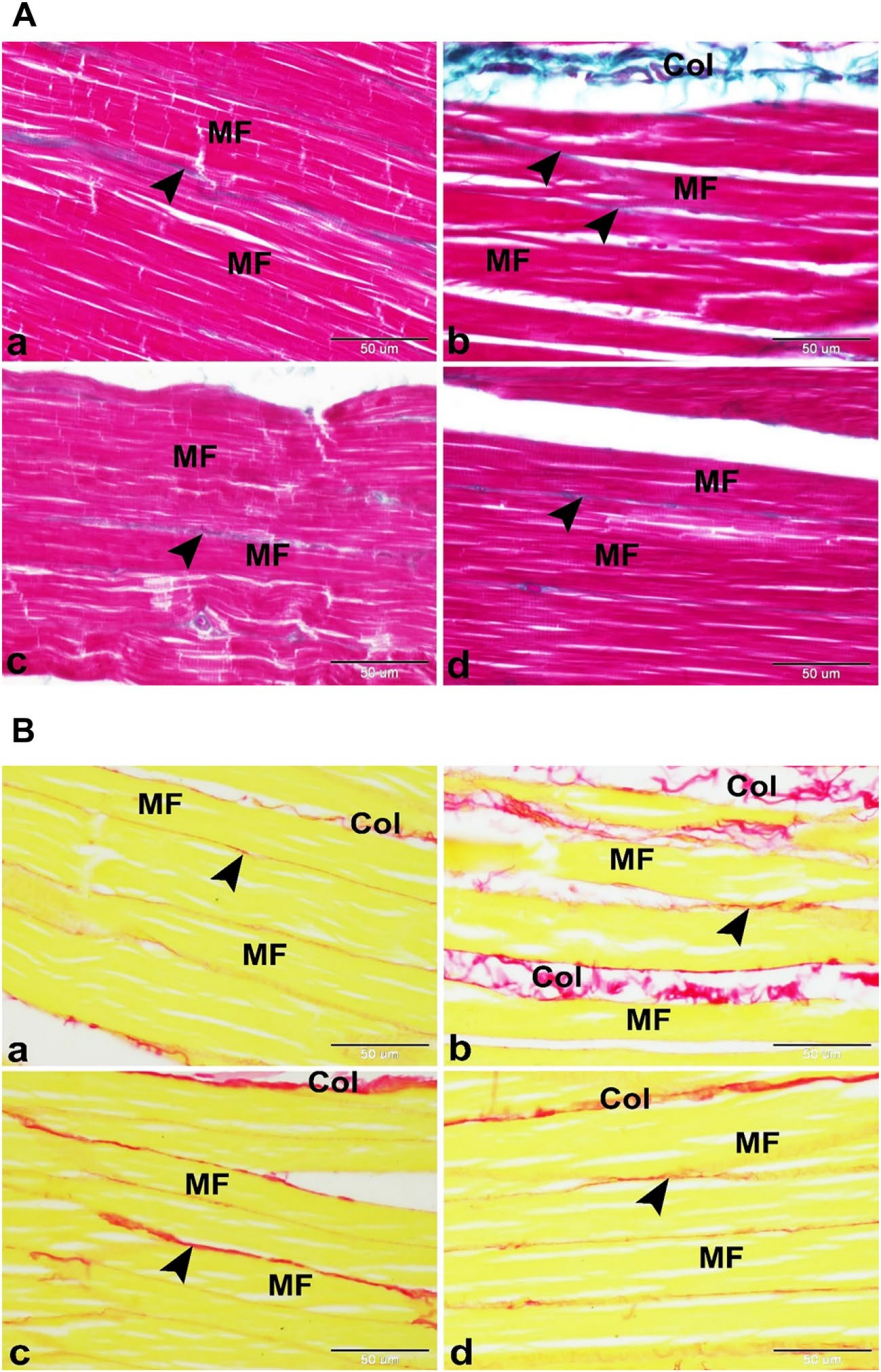
(**A**) Photomicrographs showing minimizing of the myotoxic side effects of SV by using microsponges formulations in rats. (**a**) Control group showing the normal architecture of the skeletal muscle in rat. The skeletal muscle was formed of several parallel elongated cylindrical muscle fibers (MF) with few amounts of collagen fibers in the endomysium and perimysium. (**b**) Free SV group showing the myotoxic side effects of SV as increased amounts of collagen fibers in the endomysium (arrowhead) and perimysium (Col) which surround the muscle fibers (MF). (**c**) FSV-6 group showing few amounts of collagen fibers in the endomysium and perimysium (arrowhead) which surround the muscle fibers (MF). (**d**) FSV-1 group showing minimizing of the myotoxic side effects of SV. The skeletal muscle was formed of several parallel elongated cylindrical muscle fibers (MF) with few amounts of collagen fibers in the endomysium and perimysium (arrowhead). Masson’s trichrome, scale bar = 50 μm. (**B**) Photomicrographs showing minimizing of the myotoxic side effects of SV by using microsponges formulas in rats. (**a**) Control group showing several parallel elongated cylindrical muscle fibers (MF) with few amounts of mature collagen fibers in the endomysium (arrowhead) and perimysium (Col). (**b**) Free SV group showing the myotoxic side effects of SV as increased amounts of mature collagen fibers in the endomysium (arrowhead) between muscle fibers (MF) and perimysium (Col) surround muscle bundles. (**c**) FSV-6 group showing slight decrease in amounts of mature collagen fibers in the endomysium (arrowhead) between muscle fibers (MF) and perimysium (Col) surround muscle bundles. (**d**) FSV-1 group showing few amounts of mature collagen fibers in the endomysium (arrowhead) between muscle fibers (MF) and perimysium (Col) surround muscle bundles. Sirius red, scale bar = 50 μm.

**Figure 4:**
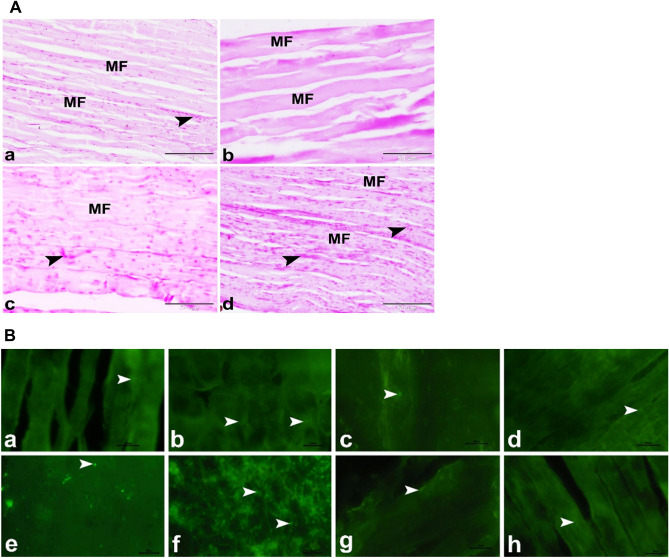
(**A**) Photomicrographs showing minimizing of the myotoxic side effects of SV by using microsponges formulas in rats. (**a**) Control group showing the normal architecture of the skeletal muscle in rat. The skeletal muscle was formed of several parallel elongated cylindrical muscle fibers (MF) with large amounts of PAS positive glycogen (arrowhead) in the sarcoplasm. (**b**) Free SV group showing the myotoxic side effects of SV as depletion of PAS positive glycogen (arrowhead) in the sarcoplasm of the wavy muscle fibers (FM). (**c**) FSV-6 group showing increased amounts of PAS positive glycogen (arrowhead) in the sarcoplasm of muscle fibers (FM). (**d**) FSV-1 group showing minimizing of the myotoxic side effects of SV. The skeletal muscle was formed of several parallel elongated cylindrical muscle fibers (MF) with large amounts of PAS positive glycogen (arrowhead) in the sarcoplasm. PAS, scale bar = 50 μm. (**B**) Photomicrograph of GR (**a–d**) and SOD2 (**e–h**) immunostaining in the skeletal muscles; (**a,e**) Control group, (**b,f**) Free SV group, (**c,g**) FSV-6 group and (**d,h**) FSV-1 group showing that GR immuno-expression (arrowheads) was nearly similar in all experimental groups, while SOD2 immuno-expression (arrowheads) was significantly increased in SV group and it was significantly decreased in FSV-6 group and FSV-1 group compared to control group, scale bar = 20 μm.

Histological examinations of the control group showed the skeletal muscle’s standard structure in rats. The skeletal muscle comprised several parallel elongated cylindrical muscle fibers with multiple flattened peripherally located nuclei beneath the sarcolemma and acidophilic sarcoplasm (Fig. 2A-a). The skeletal muscle fiber showed clear transverse striation formed of alternating dark and light bands (Fig. 2B-a,C-a). The muscle fibers are separated from each other by a few amounts of mature collagen fibers of endomysium and arranged in groups, each surrounded by a few quantities of mature collagen fibers of perimysium (Fig. 3A-a,B-a). The sarcoplasm of the muscle fibers contained large amounts of PAS-positive glycogen granules (Fig. 4A-a). On the other hand, the free SV group showed the myotoxic side effects of free SV as; degenerated muscle fibers with deeply stained acidophilic sarcoplasm, pyknotic nucleus, hemorrhage, and leucocytic infiltration (Fig. 2A-b).

The skeletal muscle fibers in the free SV group also displayed absence or ill clear transverse striation (Fig. 2B-b,C-b). Similar findings were also obtained with rosuvastatin^26^. Muscular inflammatory cell infiltration is a normal healing response secondary to muscular injury^17^. The skeletal muscles of the free SV group displayed increased amounts of mature collagen fibers in the endomysium between muscle fibers and perimysium surrounding muscle bundles (Fig. 3A-b,B-b).

The sarcoplasm of the muscle fibers showed a decline in PAS-positive glycogen granules (Fig. 4A-b). Mechanisms for prospecting comprise intracellular loss of essential metabolites and destabilization of cell membranes, influencing risen cytotoxicity^9^. The most popular hypothesis for statin myopathy is the loss of intramuscular CoQ10 resulting in mitochondria dysfunction, later abnormal muscle energy metabolism, symptoms^6^, and the inheritable association with exercise-gained muscle damage and muscle injury threat^27^. The mechanism of statins-induced skeletal muscle damage is ambiguous, but current findings propose that statins lower the output of small regulatory proteins, which are essential for maintaining myocytes^28^.

Other ways of myopathy induced by statins involve lower cholesterol synthesis and manufacture of prenylated proteins, lowered dolichols, and enhanced atrogin-1 expression^29^. Contrary to the findings of the free SV group, the FSM-6 group showed a slight minimization of the myotoxic side effects of SV compared to the control group. The sarcoplasm was still deeply stained (Fig. 2A-c) and showed ill clear transverse striation (Fig. 2B-c,C–c). The muscle fibers showed few amounts of collagen in the endomysium and perimysium (Fig. 3A-c,B-c) and increased amounts of the PAS-positive glycogen granules (Fig. 4A-c). This indicated that the FSM-6 formula still adversely affected the skeletal muscles. At the same time, the FSM-1 group minimized the myotoxic side effects of SV compared to the control. The skeletal muscle showed the standard histological structure of the skeletal muscle in the rat (Fig. 2A-d). The skeletal muscle fiber showed clear transverse striation formed of alternating dark and light bands (Fig. 2B-d,C-d). The muscle fibers showed few collagen fibers in the endomysium and perimysium (Fig. 3A-d,B-d) and increased amounts of the PAS-positive glycogen granules (Fig. 4A-d). Our study revealed that the expression of the tissue oxidative stress marker GR was significantly increased in the Free SV group (Fig. 4B-b) and decreased in the FSM-6 group (Fig. 4B-c) and FSM-1 group (Fig. 4B-d) compared to the control group (Fig. 4B-a).

In contrast, the tissue expression of oxidative stress marker SOD2 was nearly unaffected in all experimental groups (Fig. 4B-e–h). SOD2 isoforms are in the matrix of mitochondria. SOD2 catalyzes the dismutation of O_2_ − to H_2_O_2_ and maintains the redox balance by diffusing the superoxide^30^. Superoxide radical anion (O_2_,‒) and other reactive oxygen species are generated throughout respiration. However, increases in SOD2 expression enhance oxidative stress, indicating that there may be a prooxidant role for SOD2^31^. It was demonstrated that the fusion of iron by the mitochondrial superoxide dismutase (SOD2) generates a prooxidant peroxidase in the mitochondria of human cells and mice. FeSOD2 formation resulted in mitochondrial disturbance and oxidative stress^31^, which may be the leading cause of muscular damage.

### Biochemical studies

**Figure 5:**
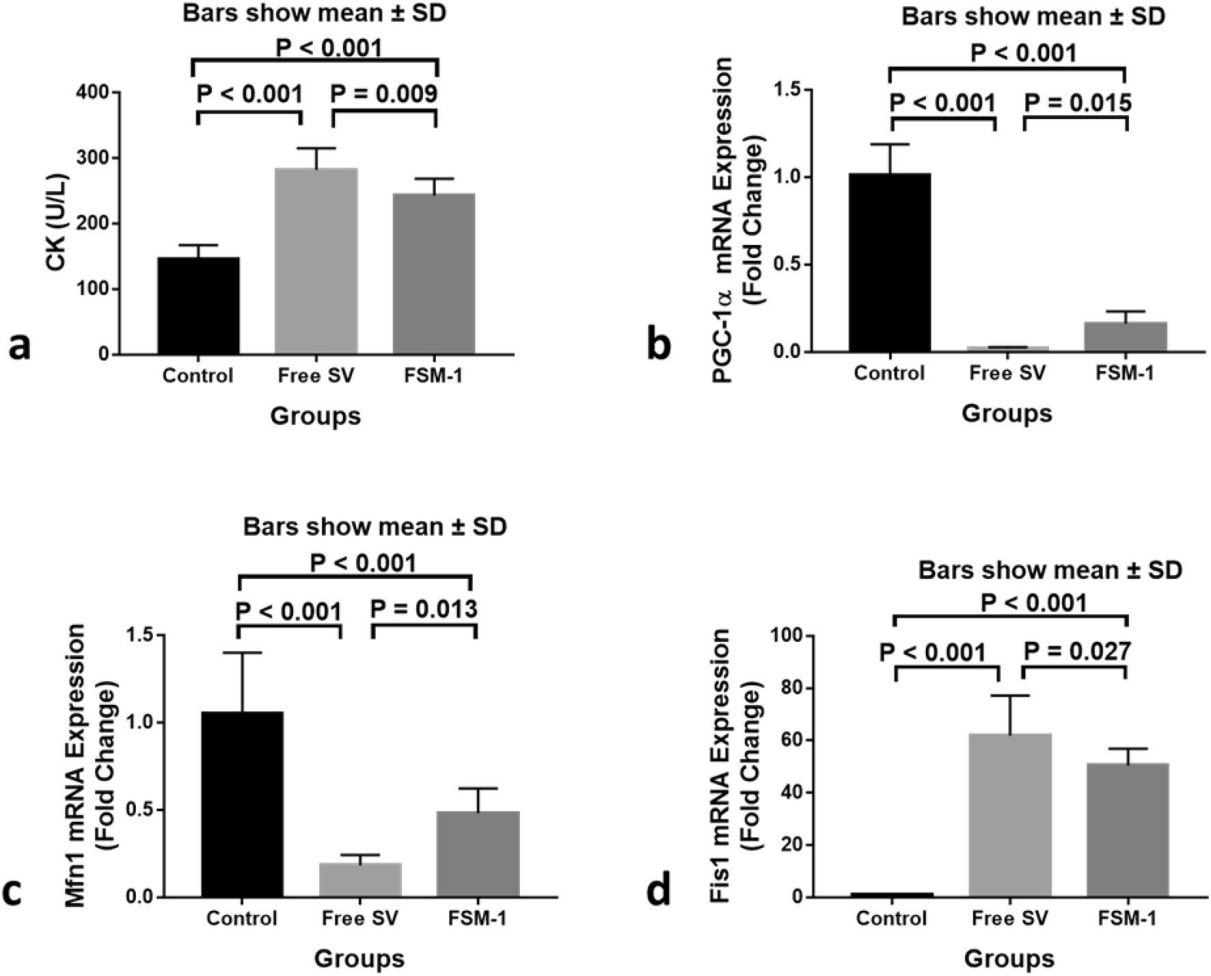
Serum levels of CK (**a**) and Gene expression levels of PGC-1α (**b**), MFn1 (**c**), and Fis1 (**d**) of the studied groups (n = 8). Data are given as mean ± SD. P < 0.05 is considered significant using a one‐way analysis of variance with LSD post hoc test (n is number/group).

Histological studies revealed the superiority of FSM-1 on FSM-6 in minimizing the myotoxic side effects of SV, so that no further biochemical studies will be carried out on the FSM-6 group. The level of creatine kinase (CK) was found to be significantly higher in the free SV group compared to the control group (P < 0.001). However, in comparison with the free SV group (P = 0.009), it was significantly lower in the FSM-1 group, although still more significant than the control group (P < 0.001; Fig. 5a). These findings are compatible with those of Chogtu et al.^32^ who found significantly higher CK levels in the statin-treated rat group. CK level was significantly decreased in the FSM-1 group compared to the free SV group but still higher than that in the control group. This finding implies that FSM-1 can somewhat mitigate the damage caused by free SV to skeletal muscle cells. The skeletal and cardiac muscles contain CK, considered the best marker for identifying and monitoring skeletal muscle diseases^27^. Nakahara et al. had previously discovered that statin-induced changes in cell membranes could lead to CK leakage before the onset of muscle symptoms^33^.

### PGC-1α, FMN1, and Fis1 gene expression in skeletal muscles

**Figure 6:**
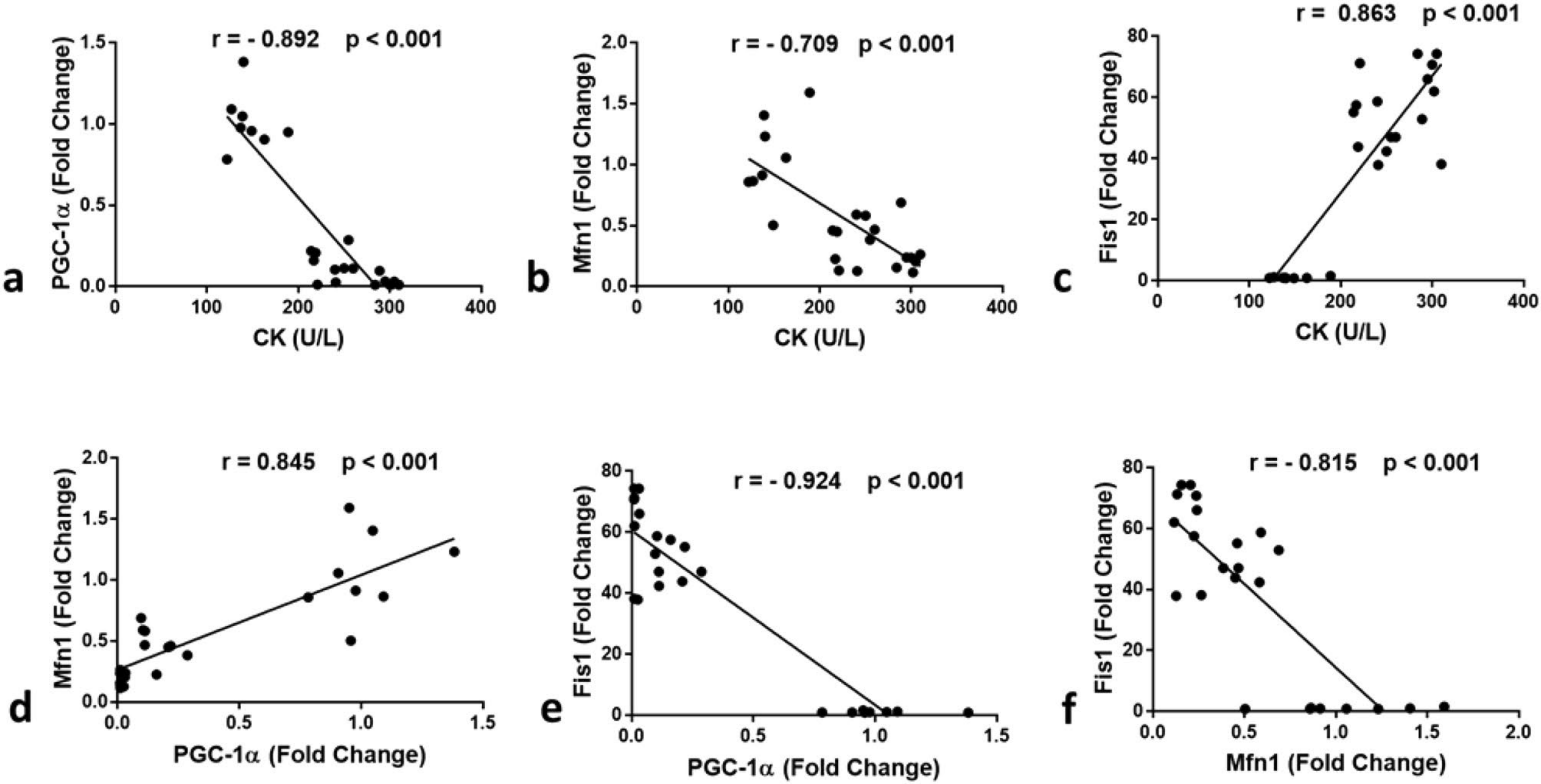
Correlation between (**a**) CK and PGC-1α; (**b**) CK and Mfn1; (**c**) CK and Fis1; (**d**) PGC-1α and Mfn1; (**e**) PGC-1α and Fis1; (**f**) Mfn1 and Fis1. Significant positive correlation was found between (**c**) CK and Fis1 (r = 0.863, P < 0.001), (**d**) PGC-1α and Mfn1 (r = 0.845, P < 0.001). On the other hand, significant negative correlation was found (**a**) CK and PGC-1α (r =  − 0.892, P < 0.001), (**b**) CK and Mfn1 (r =  − 0.709, P < 0.001), (**e**) PGC-1α and Fis1 (r =  − 0.924, P < 0.001), (**f**) Mfn1 and Fis1 (r =  − 0.815, P < 0.001). r: Pearson correlation factor. The Correlations are analyzed between the studied parameters in the control, Free SV, and FSM-1 groups (n = 24).

Our current study clarified that PGC-1α (P < 0.001) and Mfn1 (P < 0.001) gene expression was significantly downregulated in the free SV group compared to the control group. However, their expression level was significantly upregulated in the FSM-1 group compared to the free SV group (P = 0.015, P = 0.013 respectively) but still lower than those in the control group (P < 0.001 for each, Fig. 5b,c). These results demonstrated that PGC-1α gene expression was much lower in the free SV group, which was consistent with a previous study by Hardjo and Kadir^34^. Vaughan et al.^35^ found that statins reduced PGC1α gene expression in human rhabdomyosarcoma cultured cells. Statin-myopathy has been linked to altered mitochondrial function and morphology in several clinical, animal, and cell culture studies. Statins cause a decrease in mitochondrial content/volume due to decreased mitochondrial biogenesis, which is regulated by several signaling molecules and transcriptional co-activators, including PGC1α^36^. However, in our study, the PGC1α expression level was significantly upregulated in the FSM-1 group compared to the free SV group but still lower than in the control group. This outcome could point to the ability of FSM-1 being able to reduce statin-induced myotoxicity in skeletal muscle via boosting PGC1 α expression. Meanwhile, Fis1 gene expression was significantly upregulated in the free SV group compared to the control group (P < 0.001), but it was downregulated in the FSM-1 group compared to the free SV group (P = 0.027) but still higher than those in the control group (P < 0.001, Fig. 5d). Mitochondria occur in skeletal muscle as dynamic networks constantly rebuilding through fusion and fission. These processes, known as mitochondrial dynamics, are critical for mitochondrial function. Mitofusin proteins (Mfn1) in the outer mitochondrial membrane govern mitochondrial fusion. In conjunction with fission protein 1 (Fis1), dynamin-related protein 1 (Drp1), a cytosolic protein that translocates to the outer surface of mitochondria when activated^37^. Mitochondrial quality control systems maintain mitochondrial function by isolating and removing damaged mitochondria through selective fusion, fission, and mitochondrial autophagy (mitophagy)^8^. The current study looked at the influence of SV on the dynamic behavior of mitochondria in rat muscle cells, including the balance between fusion and fission, to figure out what causes SAMS. Mfn1 gene expression was significantly lower in the free SV group, whereas FSM-1 treatment considerably raised them; Fis1 gene expression was significantly higher in the free SV group but decreased in the FSM-1 group. This was in line with prior research that linked statin medication to mitochondrial dysfunction^38,39^. Statin therapy has been linked to skeletal muscle mitochondrial dysfunction, which could explain several muscular myopathies, such as weakness and fatigue, that statin users have noticed^20^. Yüksel and Baykara^21^ discovered no differences in mitochondrial function between control and simvastatin-treated mice, which contradicts our findings. Our results also demonstrate that PGC-1α was positively correlated with Mfn1and negatively correlated with CK and Fis1. These findings go hand in hand with Garnier et al.^40^. A block in fusion could result in smaller mitochondria with either missing mitochondrial DNA or enriched with mutant mitochondrial DNA. In contrast, downregulation of Mfn1 has been found to result in fragmented mitochondria with drastically reduced oxygen consumption and ATP synthesis. Mitochondrial fragmentation is caused by Fis1 overexpression^41^. All correlations are summarized in (Fig. 6). The correlations between the different parameters were done using Pearson's correlation, where a significant positive correlation was observed between CK and Fis1 (r = 0.863, P < 0.001), PGC-1α, and Mfn1 (r = 0.845, P < 0.001) (Fig. 6c,d).

However, a significant negative correlation was found between CK and PGC-1α (r =  − 0.892, P < 0.001), CK and Mfn1(r =  − 0.709, P < 0.001), PGC-1α and Fis1 (r =  − 0.924, P < 0.001), Mfn1 and Fis1 (r =  − 0.815, P < 0.001), (Fig. 6a,b,e,f). Few studies used novel delivery systems, that were differ from our design, to alleviate SV myotoxicity. A study performed by (Jones et al.) in which hyaluronic acid derived nanoparticle loaded with SV were prepared and reduced the disruption in cytoskeleton and reductions in metabolic activity in a tissue engineered skeletal muscle^42^. In another study, solid lipid nanoparticles of SV developed by (Abo-Zalam et al.) minimized the SV-associated myopathy in quadriceps muscles of rats. In our work, the SV microsponges showed fewer myotoxic side effects even if high SV dose (20 mg/kg/day) was used^43^.

## Conclusion

Microsponges with SV had been produced successfully using the emulsion solvent evaporation method. Changing the preparation temperature, the Mg stearate concentration or even the volume internal phase had an insignificant effect on the %EE, the particle size, or the %CDR of the prepared microsponges formulations. The %CDR of FSM-1 was significantly higher than that of the free drug in the dissolution media, which indicates solubility enhancement by the selected SV microsponges formulation. Although FSM-1 showed the highest %CDR, histological examination of skeletal muscles demonstrated that it is the safest microsponges formulation on the skeletal muscles compared to the myotoxic side effects of the free SV, which may be due to the ability of microsponges to control the release of drugs. The safety of SV microsponges was further confirmed by assessment of CK levels, gene expression of Mfn1 & Fis1, and PGC-1α in free SV and FSM-1 groups compared with the control group. Thus, a delivery system capable of enhancing the solubility and controlling the release of SV could dramatically decrease the myotoxic drawbacks of SV.

## Data Availability

The datasets analysed during the current study available from the corresponding author on reasonable request.
